# Detecting biomarkers by dynamic nuclear polarization enhanced magnetic resonance

**DOI:** 10.1093/nsr/nwae228

**Published:** 2024-06-29

**Authors:** Shizhen Chen, Lei Zhang, Sha Li, Yaping Yuan, Bin Jiang, Zhongxing Jiang, Xu Zhang, Xin Zhou, Maili Liu

**Affiliations:** State Key Laboratory of Magnetic Resonance and Atomic and Molecular Physics, National Center for Magnetic Resonance in Wuhan, Wuhan Institute of Physics and Mathematics, Innovation Academy for Precision Measurement Science and Technology, Chinese Academy of Sciences, Wuhan 430071, China; University of Chinese Academy of Sciences, Beijing 100049, China; School of Biomedical Engineering, Hainan University, Haikou 570228, China; State Key Laboratory of Magnetic Resonance and Atomic and Molecular Physics, National Center for Magnetic Resonance in Wuhan, Wuhan Institute of Physics and Mathematics, Innovation Academy for Precision Measurement Science and Technology, Chinese Academy of Sciences, Wuhan 430071, China; University of Chinese Academy of Sciences, Beijing 100049, China; State Key Laboratory of Magnetic Resonance and Atomic and Molecular Physics, National Center for Magnetic Resonance in Wuhan, Wuhan Institute of Physics and Mathematics, Innovation Academy for Precision Measurement Science and Technology, Chinese Academy of Sciences, Wuhan 430071, China; University of Chinese Academy of Sciences, Beijing 100049, China; State Key Laboratory of Magnetic Resonance and Atomic and Molecular Physics, National Center for Magnetic Resonance in Wuhan, Wuhan Institute of Physics and Mathematics, Innovation Academy for Precision Measurement Science and Technology, Chinese Academy of Sciences, Wuhan 430071, China; University of Chinese Academy of Sciences, Beijing 100049, China; State Key Laboratory of Magnetic Resonance and Atomic and Molecular Physics, National Center for Magnetic Resonance in Wuhan, Wuhan Institute of Physics and Mathematics, Innovation Academy for Precision Measurement Science and Technology, Chinese Academy of Sciences, Wuhan 430071, China; University of Chinese Academy of Sciences, Beijing 100049, China; State Key Laboratory of Magnetic Resonance and Atomic and Molecular Physics, National Center for Magnetic Resonance in Wuhan, Wuhan Institute of Physics and Mathematics, Innovation Academy for Precision Measurement Science and Technology, Chinese Academy of Sciences, Wuhan 430071, China; University of Chinese Academy of Sciences, Beijing 100049, China; State Key Laboratory of Magnetic Resonance and Atomic and Molecular Physics, National Center for Magnetic Resonance in Wuhan, Wuhan Institute of Physics and Mathematics, Innovation Academy for Precision Measurement Science and Technology, Chinese Academy of Sciences, Wuhan 430071, China; State Key Laboratory of Magnetic Resonance and Atomic and Molecular Physics, National Center for Magnetic Resonance in Wuhan, Wuhan Institute of Physics and Mathematics, Innovation Academy for Precision Measurement Science and Technology, Chinese Academy of Sciences, Wuhan 430071, China; University of Chinese Academy of Sciences, Beijing 100049, China; School of Biomedical Engineering, Hainan University, Haikou 570228, China; State Key Laboratory of Magnetic Resonance and Atomic and Molecular Physics, National Center for Magnetic Resonance in Wuhan, Wuhan Institute of Physics and Mathematics, Innovation Academy for Precision Measurement Science and Technology, Chinese Academy of Sciences, Wuhan 430071, China; University of Chinese Academy of Sciences, Beijing 100049, China

**Keywords:** MRI, dynamic nuclear hyperpolarization, biomarker, responsive agent

## Abstract

Hyperpolarization stands out as a technique capable of significantly enhancing the sensitivity of nuclear magnetic resonance (NMR) and magnetic resonance imaging (MRI). Dynamic nuclear polarization (DNP), among various hyperpolarization methods, has gained prominence for its efficacy in real-time monitoring of metabolism and physiology. By administering a hyperpolarized substrate through dissolution DNP (dDNP), the biodistribution and metabolic changes of the DNP agent can be visualized spatiotemporally. This approach proves to be a distinctive and invaluable tool for non-invasively studying cellular metabolism *in vivo*, particularly in animal models. Biomarkers play a pivotal role in influencing the growth and metastasis of tumor cells by closely interacting with them, and accordingly detecting pathological alterations of these biomarkers is crucial for disease diagnosis and therapy. In recent years, a range of hyperpolarized DNP molecular bioresponsive agents utilizing various nuclei, such as ^13^C, ^15^N, ^31^P, ^89^Y, etc., have been developed. In this context, we explore how these magnetic resonance signals of nuclear spins enhanced by DNP respond to biomarkers, including pH, metal ions, enzymes, or redox processes. This review aims to offer insights into the design principles of responsive DNP agents, target selection, and the mechanisms of action for imaging. Such discussions aim to propel the future development and application of DNP-based biomedical imaging agents.

## Teaser

This review summarizes recent research progress on detecting biomarkers in the tumor microenvironment using dynamic nuclear polarization techniques and discusses future directions of development in this field.

## INTRODUCTION

A biomarker is a characteristic that is objectively measured and evaluated as an indicator of normal biological processes, pathogenic processes, or pharmacological responses to an intervention. Biomarkers can play a critical role in identifying the disease state, identifying factors contributing to disease progression, and predicting and monitoring response to treatment. The identification of biomarkers that can be used as diagnostics or predictors of treatment response is an important step in this direction. Magnetic resonance (MR) techniques are the most versatile and powerful analytical tools for biomarker detection [1–4]. Nevertheless, a significant challenge of MR techniques lies in their relatively low sensitivity. This inherent limitation significantly restricts the applicability of MR techniques in detecting molecules other than water, rapid dynamic processes, and crucial markers for an array of diseases [5]. The intensity of the MR signal is directly proportional to the nuclear spin polarization reflecting the population difference among nuclear spin Zeeman states [6]. At a magnetic field of 3 Tesla and a temperature of 298 K, the thermal polarization of ^1^H is merely 10 ppm (parts per million). In practical terms, this means that out of every million protons, only 10 contribute to the net NMR signal. Furthermore, the gyromagnetic ratio of ^13^C is only a fourth of that of ^1^H, resulting in a lower thermal polarization of less than 3 ppm. Over the years, major MR vendors such as GE, Siemens, Philips, and United Imaging have invested efforts in enhancing nuclear polarization by developing higher magnetic field strengths, reaching up to 14 T for humans and 21 T for small animals [7]. However, these advancements yield limited improvements in sensitivity and come with prohibitively high costs. For example, increasing the field strength from 3 to 14 T results in less than a fivefold increase in polarization.

Hyperpolarization techniques, designed to establish a non-equilibrium distribution of nuclear spins, present a groundbreaking solution to sensitivity challenges [8]. Predominantly, these techniques include parahydrogen-induced polarization (PHIP), dynamic nuclear polarization (DNP), and spin exchange optical pumping (SEOP). PHIP is a low-cost method that involves the transfer of polarization from *p*-H_2_ onto other nuclei of interest following a chemical reaction under typical conditions [9]. Currently, this technology has found numerous applications in the fields of catalysis research [10]. SEOP entails the transfer of polarization from electrons to nuclei of interest through laser irradiation, commonly employed for hyperpolarizing noble gases like ^129^Xe and ^3^He [11]. Hyperpolarizated ^129^Xe MRI has emerged as a powerful tool for evaluating ventilation and gas exchange within the lungs [12,13]. Among these, DNP, particularly noteworthy for hyperpolarizing various molecules in liquid and solid states, stands out as the primary method for producing hyperpolarized responsive agents [14–17].

DNP operates by transferring polarization from unpaired electrons to coupled nuclei through microwave irradiation, a concept initially proposed by Overhauser and later experimentally proved by Carver and Slichter [18,19]. Considering the electron's gyromagnetic ratio being 660 times that of ^1^H and 2600 times that of ^13^C, the maximum theoretical enhancement via DNP can achieve 660-fold for ^1^H and 2600-fold for ^13^C. When DNP is conducted at liquid helium temperatures (1–1.4 K) and high magnetic fields (3.35–11.8 T), followed by rapid signal acquisition at room temperature, enhancements can surpass 10 000-fold relative to signals obtained under thermal equilibrium [20]. To date, ^13^C polarization has reached up to 70%, exceeding its thermal polarization of ^13^C at a magnetic field strength of 1 000 000 T at room temperature [21].

**Scheme 1.**
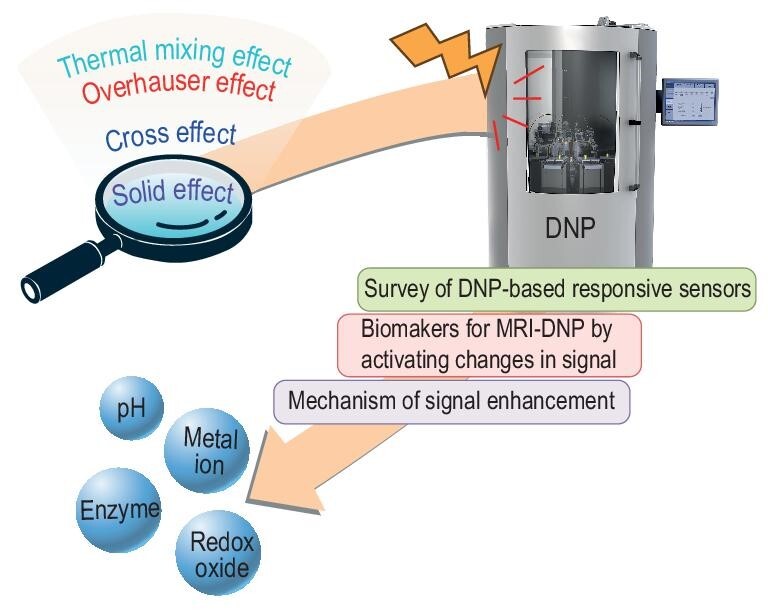
Towards utilizing hyperpolarized bioresponsive agents for functional molecular imaging with magnetic resonance.

The DNP method has been instrumental in hyperpolarizing various nuclei, such as ^1^H, ^13^C, ^15^N, ^17^O, ^19^F, and ^31^P, leading to the development of a series of hyperpolarized responsive probes [22–27]. Empowered by the DNP method, these agents can detect rapid dynamic processes and physiological information in single-scan NMR/MRI experiments or sequences of consecutive experiments with small flip angles [28–30]. This review centers on recently developed responsive agents hyperpolarized through the DNP method, emphasizing their applications in detecting biomarkers, such as pH, ions, enzymes, and redox status (Scheme 1). Hyperpolarization methods, especially DNP, are revolutionizing the landscape of NMR/MRI by substantially enhancing sensitivity and expanding their applicability in both biological and clinical research. The potential of hyperpolarized responsive agents in detecting intricate biological processes opens the door to groundbreaking advancements in science and medicine.

## THE BASIC MECHANISM OF DNP

NMR plays a pivotal role in non-invasively monitoring chemical reactions and is widely utilized in chemistry, biomedicine, and materials science. However, compared with other spectral analysis methods, the sensitivity of NMR limits its broader application. Therefore, enhancing magnetic resonance sensitivity remains a primary objective. One approach involves increasing detection sensitivity, such as by the use of cryogenic pick-up coils. Another strategy focuses on augmenting signal strength through high polarization. DNP is a technology that leverages the high polarization of electrons at thermal equilibrium to bolster atomic nuclear polarization. Compared with thermal polarization, molecular polarization can be greatly increased, significantly amplifying magnetic resonance signal intensity. The essence of DNP lies in exploiting the large gyromagnetic ratio of electrons. Electron polarization at thermal equilibrium is 660 times greater than that of protons at the same temperature. Therefore, after employing microwaves, the high electron polarization is transferred to nuclei through hyperfine interactions, enhancing nuclear polarization.

### The basis of polarization

Nuclei with a non-zero spin quantum number (*I* ≠ 0) undergo Zeeman splitting upon the application of an external magnetic field (*B*_0_), leading to the division of their energy states into 2*I* + 1 levels. The distribution of nuclear spin populations across these energy levels follows the principle of the Boltzmann distribution. The NMR signal intensity is directly related to the polarization level, defined as the population discrepancy among nuclei at distinct energy levels. Consequently, the expression for the polarization level (*P_I_*) in the context of nuclei with *I* = 1/2 can be simplified to Equation (1)


(1) \begin{eqnarray*}
{{P}_I} = \tanh \left( {\gamma \hbar {{B}_0}/2{{k}_{\mathrm{B}}}T} \right)
\end{eqnarray*}

where *γ* represents the gyromagnetic ratio of the proton, $\hbar$ is the reduced Planck's constant, *B*_0_ stands for the magnetic field strength, *k*_B_ denotes the Boltzmann constant, and *T* signifies the temperature. Disrupting the Boltzmann thermal equilibrium, hyperpolarization techniques amplify the difference in the population distribution among nuclear spin energy states, resulting in an enhanced NMR signal. The DNP technique has emerged as an effective hyperpolarization method, achieving signal enhancement of more than four orders of magnitude. This technique has been successfully applied to hyperpolarize various nuclei, including ^1^H, ^13^C, ^15^N, ^19^F, ^31^P, ^89^Y, and others.

### Enhancement mechanism of DNP

DNP is a hyperpolarization technique enhanced by polarization transfer from electron to nuclei. The electron energy levels are saturated through microwave irradiation, and the coupling interactions between electrons and nuclei polarize the distribution of pertinent nuclear energy levels, significantly amplifying the population difference among nuclei at distinct energy levels. This process results in a high-intensity NMR signal with enhanced polarization. The DNP polarization transfer mechanism encompasses four mechanisms: Overhauser effect, solid effect, thermal mixing effect, and cross effect [6,16,18,31–33]. The following sections briefly introduce these four polarization enhancement mechanisms.

#### Overhauser effect

**Figure 1.**
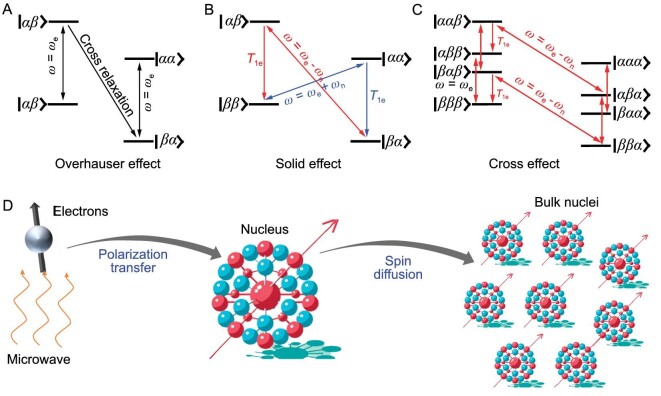
Schematic representation of Overhauser effect (A), the negative (green) and positive (blue) enhancement of solid effect (B) with four-energy-level ($| { {\beta \alpha } \rangle } $,$| { {\beta \beta } \rangle } $,$| { {\alpha \alpha } \rangle ,} $ and $| { {\alpha \beta } \rangle } $) system, the negative enhancement of cross effect (C) with the three-spin system of eight-energy-level ($| {\beta {\beta \alpha } \rangle } $,$| { {\beta \beta \beta } \rangle } $,$| {\beta {\alpha \alpha } \rangle } $,$| { {\beta \alpha \beta } \rangle } $,$| { {\alpha \beta \alpha } \rangle } $,$| { {\alpha \beta \beta } \rangle } $,$| { {\alpha \alpha \alpha } \rangle ,} $ and $| { {\alpha \alpha \beta } \rangle } $) system at thermal equilibrium (D). The microwave-polarized electron spin is first transferred to the nearby nucleus spin and then propagated to the bulk nuclei through spin diffusion in the polarization transfer process.

The Overhauser effect is applicable to samples such as liquids, metals, and organic conductors containing abundant free electrons. Due to the rapid movement of molecules or the presence of free electrons, all nuclear spins in the sample can directly interact with the electron spins to realize the transfer of electrons to the nucleus [19]. In 1953, the first attempt to transfer electron polarization to nuclear spins was based on the Overhauser effect [18]. Figure 1A illustrates a four-energy-level system ($| { {\beta \alpha } \rangle } $,$| { {\beta \beta } \rangle } $,$| { {\alpha \alpha } \rangle ,} $ and $| { {\alpha \beta } \rangle } $) comprising a free electron (*I* = 1/2) and a nucleus (*I* = 1/2) under a magnetic field. When microwave irradiation with frequency ${{\omega }_e}\ $is applied to the system, the electron energy levels are excited, transitioning to a saturation state. This leads to an equal population of $| { {\alpha \beta } \rangle } $ and $| { {\beta \beta } \rangle } $, as well as $| { {\alpha \alpha } \rangle } $ and $| { {\beta \alpha } \rangle } $.

Rapid cross-relaxation between $| { {\alpha \beta } \rangle } $ and $| { {\beta \alpha } \rangle } $ facilitates the swift attainment of the Boltzmann distribution, significantly augmenting the population difference between $| { {\alpha \beta } \rangle } + | { {\beta \beta } \rangle } $ and $| { {\alpha \alpha } \rangle } + | { {\beta \alpha } \rangle } $. Consequently, there is a marked enhancement in the nuclear spin polarization. The primary application of the Overhauser effect is observed at low fields, where the electron-nuclear interaction is time-dependent, and its significance is most pronounced when the time scale closely aligns with $\omega _e^{ - 1}$.

#### Solid effect

Solid effects are evident when unpaired electrons are stationary within the lattice. Consequently, the electron-nuclear interaction primarily manifests as a dipole-dipole interaction, which is time-independent and considerably weaker than the external magnetic field. This interaction can be perceived as a perturbation of the Zeeman energy level. As a result, a mixed-state description becomes necessary when describing the wave function of the four-energy-level system of the electron and nucleus.

As a result of this mixed state, nominally forbidden zero-quantum transitions between $| { {\alpha \beta } \rangle } $ and $| { {\beta \alpha } \rangle } $ and double-quantum transitions between $| { {\alpha \alpha } \rangle } $ and $| { {\beta \beta } \rangle } $ become permissible under effective microwave irradiation, as illustrated in Fig. 1B. When microwave irradiation with frequency $\omega \ = \ {{\omega }_e} - {{\omega }_n}$ is applied, zero-quantum transitions from $| { {\beta \alpha } \rangle } $ to $| { {\alpha \beta } \rangle } $ can be excited. Since the relaxation rate of the electron is significantly faster than that of the nucleus, the nucleus remains in the excited state while the electron returns to thermal equilibrium. This results in a notable increase in the polarization of nuclei, leading to a negative enhancement effect. Similarly, when microwave irradiation with frequency $\omega \ = \ {{\omega }_e} + {{\omega }_n}$ is applied, double-quantum transitions from $| { {\beta \beta } \rangle } $ to $| { {\alpha \alpha } \rangle } $ can be excited, yielding a positive enhancement effect.

#### Cross effect

The cross effect, considered the most potent enhancement mechanism of DNP in high fields, encompasses both electron-electron and electron-nuclear spin coupling. The intricacies of the cross-effect enhancement mechanism are elucidated through a three-spin system involving two electrons and a nucleus, as depicted in Fig. 1C. By applying microwave irradiation with frequency ${{\omega }_e}$, an electron transition to a high energy level is induced. Subsequent to the electron's relaxation, releasing a microwave of frequency close to $\omega \ = \ {{\omega }_e} + {{\omega }_n}$, the spin directions of the remaining electron and the nucleus undergo simultaneous flips, resulting in either a double-quantum transition or a zero-quantum transition. The outcome is the acquisition of positive-enhanced nuclear polarization or negative-enhanced nuclear polarization, facilitated by the substantially faster relaxation rate of electrons compared to the nucleus.

#### Thermal mixing effect

The main feature of the thermal mixing effect is similar to the cross effect, with a stronger coupling interaction between electron spins. The system is conceptualized as three interacting parts, where spin temperature depicts the spin states, and polarization transfers resemble heat exchange between these parts. These three interacting parts, which delineate the multiple electron-nuclear spin interactions, are the electron Zeeman system (EZS), the electron dipolar system (EDS), and the nuclear Zeeman system (NZS) [34]. In thermal equilibrium, the spin temperature of all three systems aligns with the lattice temperature. The application of microwave irradiation at the frequency *ω* = *ω_e_* decreases the spin temperature of the EDS. Subsequently, the spin temperature of the NZS decreases through electron-electron–nuclear energy exchange in the thermal contact between EDS and NZS. This intricate interplay results in enhanced nuclear polarization. DNP transfer occurs via two distinct pathways: one transfers electron polarization to its adjacent central core (Fig. 1D), and the other transfers the polarization from the central core to the bulk core [35,36].

## SENSORS FOR MRI-DNP BY ACTIVATING CHANGES IN SIGNAL

Through the development of these mechanisms and technologies, the DNP method has found widespread application across diverse fields such as material science, protein analysis, and the development of NMR/MRI agents, resulting in the evolution of specialized DNP techniques, including solid-state NMR (MAS DNP) and liquid-state NMR (Overhauser DNP or dissolution DNP) [37,38]. MAS DNP is primarily employed for characterizing proteins and materials under solid-state conditions, while Overhauser DNP facilitates *in situ* polarization of solutions at room temperature, primarily for theoretical studies in liquid-state DNP. A standout technique among these is dissolution DNP (dDNP), which focuses on achieving optimal sample polarization at extremely low temperatures and then rapidly dissolving the sample after DNP, preserving a significant portion of the polarization [39]. The dDNP experiment involves three principal steps: (1) hyperpolarization of the sample at low temperatures and a moderate magnetic field; (2) rapid dissolution of the sample to a liquid state; (3) MRS/MRI detection. During the first step, continuous microwave irradiation is applied to samples containing unpaired electrons at ultra-low temperatures, achieving a high level of nuclear polarization. Typically, organic free radicals (e.g. nitroxide or trityl radicals) serve as polarization agents (PAs), used at concentrations ∼10 mM [40].

Following the enhancement build-up process, the sample is rapidly dissolved into a heated solvent and transferred to an NMR scanner. During this process, polarization experiences a rapid decline with a time constant determined by the longitudinal nuclear relaxation *T*_1_, thereby shortening the available time window for detection. *T*_1_, also known as the longitudinal relaxation time, is a crucial parameter in MR methods. Factors affecting *T*_1_ primarily include the molecular environment, magnetic field strength, temperature, tissue type and composition, the presence of paramagnetic substances, and interactions with surrounding atoms. To mitigate *T*_1_ relaxation effects, various strategies have been proposed, including the rapid removal of radicals from the sample using reducing agents, the development of PAs linked to silica porous material that can be filtered out during dissolution, and the utilization of photo-induced non-persistent radicals that are thermally annihilated, leaving a radical-free hyperpolarized solution after dissolution [41–45].

Once radicals are removed, the *T*_1_ relaxation time of the sample itself determines the available time window for acquisition. Strategies such as functional group modifications, deuterium isotope labeling, and the use of long-lived nuclear spin states have been employed to extend the *T*_1_ relaxation time [46,47]. The application of ultrafast NMR techniques, pioneered by Frydman, enables correlation information to be obtained in a single scan, while fast imaging methods, including echo-planar spectroscopic imaging (EPSI) and single-shot three-dimensional imaging, enhance polarization efficiency and expand the available time window for data acquisition [48,49]. These advancements lay a foundation for the *in vivo* application of responsive agents.

The detection mechanism of responsive agents typically hinges on fluctuations in chemical shifts, alterations in relaxation times, or shifts in the ratio of signal intensities. Hyperpolarized nuclei exhibit chemical shifts that are markedly sensitive to their environmental factors, making them robust indicators for probing physiological parameters such as pH, metal ions, enzymes, etc. Beyond chemical shifts, changes in molecular structure and chemical milieu may also affect relaxation times [50]. Additionally, pH measurement can be facilitated by utilizing the ratio of signal intensities, as variations in chemical equilibrium under different pH conditions significantly influence this ratio.

## EXPLORATION OF RESPONSIVE SENSORS BASED ON DNP

### DNP sensors for pH

**Figure 2.**
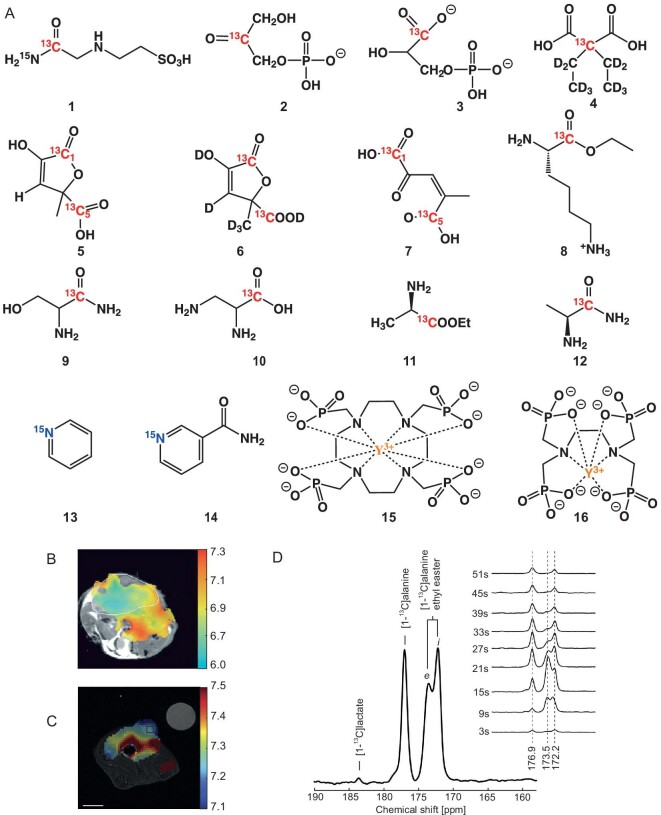
(A) The chemical structures of some reported chemical shift responsive pH agents. (B) Extracellular pH was measured by H^13^CO_3_^−^/^13^CO_2_ calculation in a subcutaneously implanted EL4 tumor mouse [55]. (C) Extracellular pH was calculated by the chemical shift difference of 1–^13^C of ZA with ^13^C labeled urea as a reference in a subcutaneous MAT B Ⅲ adenocarcinoma rat [70]. (D) ^13^C MRS metabolic process of [1–^13^C] alanine ethyl ester in rat liver [67].

pH is a critical physiological parameter that is essential for maintaining normal body functions. The occurrence and progression of diseases such as ischemia and cancer can lead to metabolic abnormalities affecting tissue pH. The precise measurement of tissue pH is of great significance for diagnosing, assessing treatment response, and determining the prognosis of diseases [51–54]. MRI is a vital noninvasive pH detection technique, but its inherently low sensitivity restricts its widespread application. dDNP significantly enhances signal strength by more than four orders of magnitude, thus facilitating noninvasive pH measurement. A summary of the pH-sensing properties of the DNP-based agent is presented in Table 1 and Fig. 2A.

**Table 1.** List of relaxation, polarization, and pH response characteristics of reported agents.

**Agents**	***T*_1_/s**	**Nuclei**	**Polarization level/%**	**p*K*_a_**	**pH range**	**Chemical shift difference/ppm**	**Ref.**
H^13^CO_3_^−^/^13^CO_2_	10.1 ± 2.9	^13^C	16	6.17	/	/	[55]
^13^C,^15^N-ACES (**1**)	25	^13^C	12.5 ± 2.7	6.56	4.5–9.0	8.4	[61]
[2–^13^C] glyceronephosphate (**2**)	/	^13^C	/	5.81	5.2–7.5	1.6	[62]
[1–^13^C] 3-phosphoglycerate (**3**)	/	^13^C	/	/	5.2–7.5	0.8	[62]
[2–^13^C,D_10_] diethylmalonic acid (**4**)	84.3 ± 1.4	^13^C	13.7 ± 0.6	7.39	5.5–9.0	3.2	[63]
[1,5–^13^C_2_] zymonic acid (**5**)	17 ± 2	^13^C	22 ± 2	6.90	5.3–8.0	4.6	[70]
[1,5–^13^C_2_,3,6,6,6-D_4_] zymonic acid (**6**)	49 ± 8	^13^C	/	/	6.4–7.4	2.3	[64]
[1,5–^13^C_2_] Z-4-methyl-2-oxopent-3-enedioic acid (**7**)	29 ± 1	^13^C	17–26	6.55	4.0–9.0	3	[65]
[1–^13^C] lysine ethyl ester (**8**)	/	^13^C	3.5	7.18	5.9–7.4	3	[66]
[1–^13^C] serine amide hydrochloride (**9**)	13.8 ± 0.4	^13^C	9.5 ± 4.9	7.35	6.4–7.6	4.2	[66]
[1–^13^C] 2,3-diaminopropionic acid hydrochloride (**10**)	18.8 ± 2.0	^13^C	7.5 ± 2.1	7.00	6.4–7.6	4.2	[66]
[1–^13^C] alanine ethyl ester (**11**)	49	^13^C	22.5	8.00	6.5–10	7.6	[67]
[1–^13^C] alaninamide (**12**)	25.2 ± 0.1	^13^C	30.5 ± 3.0	7.90	3.55–10.16	8.4	[68]
^15^N-pyridine (**13**)	41 ± 3	^15^N	1.76 ± 0.06	5.17	2.1–8.5	94	[72]
^15^N-nicotinamide (**14**)	22 ± 0.3	^15^N	1.17 ± 0.23	4.14	2.1–8.5	94	[72]
K_2_HPO_4_	29.4	^31^P	9.3	/	/	/	[73]
^89^Y-DOTP (**15**)	123	^89^Y	∼0.47	7.64	5.0–9.0	10	[71]
^89^Y-EDTMP (**16**)	90 ± 3	^89^Y	∼1.89	6.70	5.0–9.0	16	[74]

The ratiometric pH imaging method using ^13^C labeled bicarbonate hyperpolarized by dDNP has been proposed [55]. The pH values can be calculated by summing p*K*_a_ = 6.17 to the logarithm value of the concentration ratio H^13^CO_3_^−^/^13^CO_2_, with the signal intensities of H^13^CO_3_^−^ and ^13^CO_2_ measured through ^13^C MRS. Figure 2B depicts the pH map of a subcutaneously implanted EL4 tumor in a mouse. The results align with prior studies, showing that the pH values within the tumor tissue (white contour) are significantly lower than those in the surrounding normal tissue. To avoid the potential toxicity associated with the use of CsH^13^CO_3_ for the preparation of hyperpolarized bicarbonate, an indirect preparation method entailing a rapid chemical reaction between dDNP hyperpolarized ^13^C labeled α-keto acids such as pyruvic acid and H_2_O_2_ has been proposed [56]. Hyperpolarized bicarbonate with a polarization level of 10% was obtained, which was sufficient for pH mapping of phantom and isolated rat lungs. To improve the signal-to-noise ratio of ^13^CO_2_, a larger flip angle was employed to excite ^13^CO_2_ (25°) than that of H^13^CO_3_^−^ (2.78°) [57]. By combining the hyperpolarized bicarbonate indirectly prepared using synthesized [1–^13^C] 1,2-glycerol carbonate, the pH values of 7.15 ± 0.09 in tumor tissue and 7.36 ± 0.08 in normal tissue were measured in a prostate cancer mouse model, respectively. Further applications of hyperpolarized bicarbonate in pH imaging have advanced the study of disease progression in prostate cancer [58–60], showcasing its considerable promise for enhancing the management of this malignancy.

A set of ^13^C labeled, pH-responsive agents that are enhanced through DNP has been proposed, as outlined in Table 1 [61–70]. Critical design criteria include *T*_1_ relaxation time, the acid dissociation constant (p*K*a), polarization levels, chemical shift response range, and biocompatibility. The ^13^C chemical shift observed in these agents is influenced by the protonation states of proximal functional groups, determined by the pH value, such as amino groups, hydroxyl groups, phosphate groups, etc.

One example is the synthesis of ^13^C and ^15^N-labeled *N*-(2-acetamido)-2-aminoethanesulfonic acid (ACES) with a p*K*a of 6.58 and a ^13^C chemical shift difference of 8.4 ppm between pH 4.5 and 9.0 using ^13^C-labeled urea as a reference [61]. Chemical shift imaging (CSI) of ^13^C and ^15^N-labeled ACES in phantoms demonstrated accuracy within 0.1–0.2 pH units compared to the pH measured by electrodes. Another exploration involves the use of hyperpolarized phosphate metabolites, including [2–^13^C] glyceronephosphate and [1–^13^C] 3-phosphoglycerate, as pH agents with hyperpolarized pyruvate as a reference [62]. [2–^13^C] glyceronephosphate, with a p*K*a of 5.81, exhibited a larger chemical shift difference (∼1.6 ppm) than [1–^13^C] 3-phosphoglycerate (∼0.8 ppm) between pH 7.5 and 5.2. The intracellular pH could be calculated using the chemical shift difference of [2–^13^C] glyceronephosphate, reflecting catalytic activity. Additionally, hyperpolarized [2–^13^C, D_10_] diethylmalonic acid [63], with a chemical shift difference of 3.2 ppm between pH 5.5 and 9.0, a p*K*a of 7.39, and [1–^13^C, D_9_] *tert*-butanol as a reference, exhibited a long *T*_1_ relaxation time of 84.3 ± 1.4 s at 3 T.

Phantom pH imaging achieved an accuracy within 0.1 pH units when comparing the pH values obtained through the chemical shift difference of [2–^13^C, D_10_] diethylmalonic acid to electrode-measured pH values. The synthesis and hyperpolarization of [1,5–^13^C_2_] zymonic acid (ZA) resulted in a high solution polarization level of 22% ± 2%, coupled with a relatively long *T*_1_ relaxation time of 43 ± 3 s at 3 T [70]. ZA's 1–^13^C exhibited a sizable chemical shift difference (3 ppm/pH unit) within the physiological pH range, with a p*K*a of 6.90 using ^13^C-labeled urea as a reference. ZA was successfully employed for extracellular pH imaging in the mouse's bladder, kidney, and tumor regions (Fig. 2C).

The synthesis of [1,5–^13^C_2_, 3,6,6,6-D_4_] zymonic acid (ZAd) through deuteration of ZA led to reduced dipole-dipole interactions between carbons and neighboring protons, resulting in a 14% and 39% increase in *T*_1_ values for 1–^13^C and 5–^13^C of ZAd *in vitro* at 3 T compared to ZA. This deuteration enhanced the SNR by 43% and 46% in the pH imaging of a mouse tumor model at 7 T compared to ZA [64]. Furthermore, [1,5–^13^C_2_] Z-4-methyl-2-oxopent-3-enedioic acid was synthesized with a p*K*a of 6.55 and a long *T*_1_ of 29 ± 1 s *in vivo*. It was applied to mice's pH imaging of the renal cortex, renal medulla, and renal pelvis [69]. pH-sensitive derivatives of amino acids were designed and synthesized through adjustments in carbon chain length and derivatization modifications to regulate the p*K*a within the physiological range. The chemical shift of 1–^13^C of these amino acids remains constant within the physiological pH range [65–68]. For instance, the p*K*a values decreased from 8.95 for [1–^13^C] lysine to 7.18 for [1–^13^C] lysine ethyl ester [65], from 9.24 for [1–^13^C] serine to 7.35 for [1–^13^C] serine amide hydrochloride and 7.00 for [1–^13^C] 2,3-diaminopropionic acid hydrochloride [66], and from 10.00 for [1–^13^C] alanine to 8.00 for [1–^13^C] alanine ethyl ester [67] and 7.90 for [1–^13^C] alaninamide [68].

Hyperpolarized [1–^13^C] alanine ethyl ester, with a hyperpolarization level of 22.5% and a *T*_1_ relaxation time of 49 s, was utilized for pH imaging and metabolic process monitoring in the rat liver at 3 T, using [1–^13^C] alanine as a reference (Fig. 2D). Simultaneous measurements of extracellular pH 7.4 and intracellular pH 7.0 in the liver were achieved with chemically distinguishable signals at 173.5 ppm and 172.2 ppm for intracellular and extracellular [1–^13^C] alanine ethyl ester, respectively.

Beyond ^13^C-labeled pH-sensitive agents, dDNP has been employed to augment the sensitivity of ^15^N-, ^31^P-, and ^89^Y-labeled pH-responsive agents for pH measurements [71–74]. The chemical shift of ^15^N responds to the protonation of nitrogen functional groups induced by pH fluctuations. ^15^N-labeled pyridine and its derivatives exhibit chemical shift differences exceeding 88.0 ppm [72], among the most known. These pronounced chemical shift discrepancies are effective for detecting minor pH changes. However, the practical application must take into account both biocompatibility and the p*K*a value, given that the toxicity of pyridine and the p*K*a of certain pyridine derivatives, like nicotinamide with a p*K*a of 4.14, may deviate from the physiological pH range.

A series of hyperpolarized phosphates in aqueous media has been investigated [73], wherein K_2_HPO_4_ exhibited the longest *T*_1_ relaxation time of 29.4 s and a polarization level of 9.3% at 5.8 T. The immediate observation of pH change from 8.1 to 4.8 occasioned by the addition of citrate-Tris buffer was observed with hyperpolarized KH_2_PO_4_.

^89^Y-labeled compounds, ^89^Y-DOTP [71], and ^89^Y-EDTMP [74] have been proposed with p*K*a values of 7.64 and 6.70, respectively. The chemical shifts of these compounds are affected by the protonation of non-coordinating phosphonate oxygens, resulting in chemical shift differences of 10 ppm and 16 ppm over pH ranges of 5.0 to 9.0, respectively. Moreover, both ^89^Y-DOTP and ^89^Y-EDTMP exhibit relatively long *T*_1_ relaxation times of 123 s (pH 7.0) and 90 s (pH 7.0) at 9.4 T, attributed to the intrinsic *T*_1_ of ^89^Y^3+^ (*T*_1_ ≥ 600 s).

### DNP sensors for metal ions

Divalent metal ions are crucial in myriad biochemical processes, and their imbalance can contribute to diverse diseases. Therefore, developing selective, sensitive, and fast-responding sensors for metal ions detection is paramount for *in vitro* medical diagnostics. DNP boosts NMR sensitivity by orders of magnitude and is a potent technique for identifying divalent metal ions.

**Figure 3.**
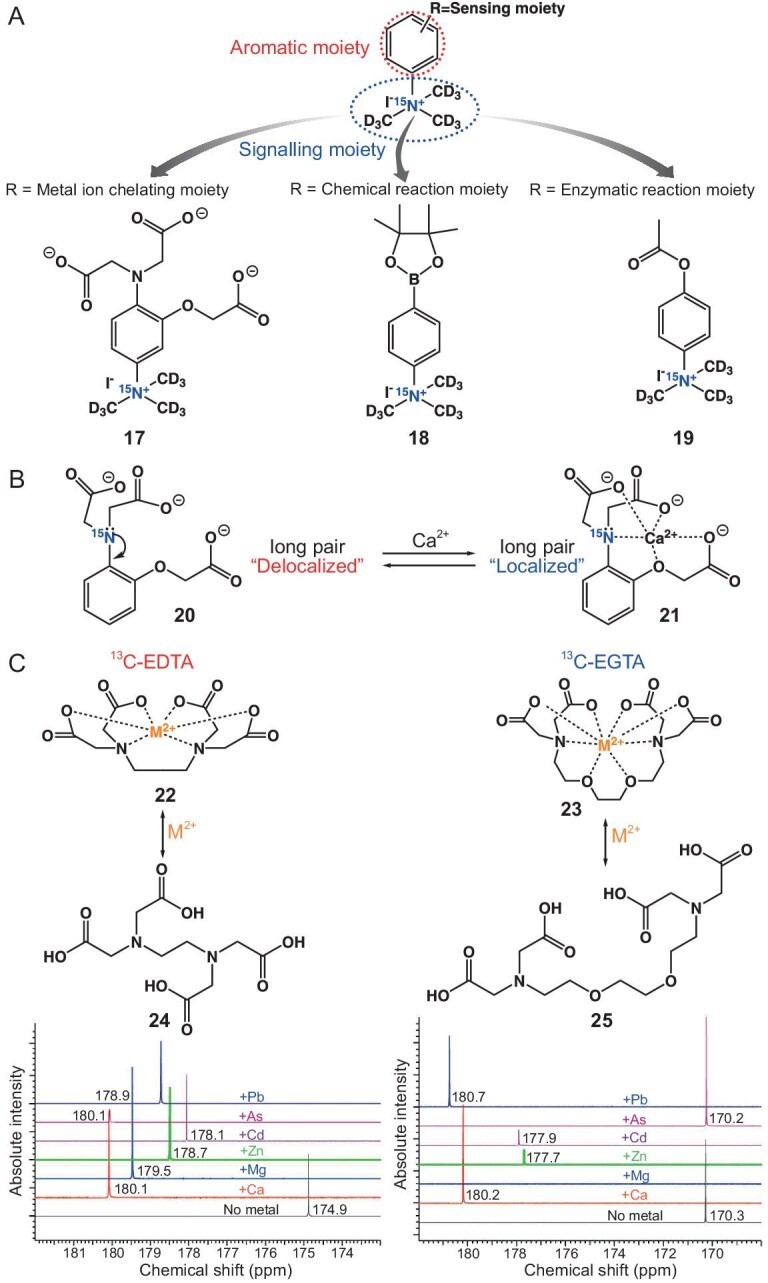
(A) Proposed [^15^N, D_9_] trimethylphenylammonium (TMPA) platform for designing hyperpolarized MR agents. Various hyperpolarized MR agents can be designed by the same strategy through straightforward derivatization of aromatic moieties. The chemical structures of agents 17–19 used in this study are shown. (B) The structure of ^15^N APTRA and schematic illustration of the mechanism to induce a sizeable ^15^N chemical shift change upon binding of Ca^2+^. (C) The structural sketches illustrate the coordination of divalent metals to the ^13^C-EDTA and ^13^C-EGTA sensors. NMR spectra obtained from both sensors showed metal-specific chemical shifts in response to divalent metals (2.2 mM of Ca^2+^, Mg^2+^, Zn^2+^, Cd^2+^, As^2+^, or Pb^2+^).

Nonaka *et al.* fabricated [^15^N, D_9_] trimethylphenylammonium (TMPA) as a promising DNP agent with an exceptionally prolonged hyperpolarization lifetime and a lengthy *T*_1_ value (816 s at 14.1 T) [75]. Through straightforward derivatization of aromatic moieties, [^15^N, D_9_] TMPA was realized to target a specific biochemical event: metal ions (Ca^2+^), reactive oxygen species (H_2_O_2_), and enzymes (carboxyl esterase) (Fig. 3A and Table 2).

**Table 2.** List of relaxation, polarization, and metal ions response characteristics of reported agents.

**Agents**	***T*_1_/s**	**Nuclei**	**Polarization level/%**	**Sensor**	**Chemical shift difference/ppm**	**Ref.**
^15^N-TMPA (**17**)	129 ± 22 (9.4 T)	^15^N	2.0	Ca^2+^	1.5	[75]
^15^N-TMPA (**18**)	444 ± 11 (9.4 T)	^15^N	2.0	H_2_O_2_	1.1	[75]
^15^N-TMPA (**19**)	536 ± 33 (9.4 T)	^15^N	2.0	Esterase	1.1	[75]
^15^N-APTRA (**20**/**21**)	37/36 (9.4 T)	^15^N	/	Ca^2+^	5.2	[76]
^13^C-EDTA (**22**/**24**)	179.98/174.79 (11.7 T)	^13^C	1–2	Ca^2+^	5.2	[77]
^13^C-EGTA (**23**/**25**)	180.13/170.34 (11.7 T)	^13^C	4–5	Ca^2+^	9.8	[77]

The originally designed [^15^N, D_9_] TMPA derivative functioned as a chemical shift-switching agent. However, the ^15^N chemical shift change upon Ca^2+^ binding was small (1.5 ppm). Subsequently, the same group delved into the electron density change of the ^15^N atom in the Ca^2+^ chelator framework [76]. They designed and synthesized hyperpolarized ^15^N labeled *o*-aminophenol-*N, N, O*-triacetic acid (APTRA). The lone pair of the ^15^N atom in APTRA was expected to delocalize into the aromatic ring without Ca^2+^ and be localized upon binding to Ca^2+^ because of the coordination. It induced a significant chemical shift change (∼5 ppm) upon Ca^2+^ binding and achieved Ca^2+^ sensing in a hyperpolarized state (Fig. 3B).

Selectively detecting multiple ions represents a multiscale functionality that provides important information. Mishra *et al.* demonstrated the effectiveness of ^13^C-EDTA and ^13^C-EGTA as hyperpolarizable multi-metal sensors [77]. Both EDTA and EGTA exhibited prolonged relaxation times (up to 15 s) and large chemical shifts (up to 10 ppm) in their carboxyl resonances upon coordination with Ca^2+^. To attain metal-specific MRI with increased sensitivity, the researchers strategically positioned ^13^C labels at the metal-coordination sites of selected chelators, amplifying their NMR signal through DNP and yielding distinct carboxyl resonances upon metal coordination. Their findings illustrated the capability of the metal-specific chemical shifts of ^13^C-EDTA and ^13^C-EGTA to differentiate between biologically essential (Ca^2+^, Mg^2+^, Zn^2+^) and toxic (Cd^2+^, As^2+^, Pb^2+^) divalent metals, enabling the determination of calcium concentration in human serum (Fig. 3C).

Diamagnetic Zn^2+^ stands out as a crucial target for imaging, given its involvement in diverse biochemical processes such as enzyme catalysis, neurotransmission, intracellular signaling, and antibiotic activity. Suh *et al.* introduced a hyperpolarized ^15^N labeled tris-(2-pyridylmethyl) amine (TPA) for the detection and quantification of Zn^2+^ [78]. The tertiary ^15^N atom in deuterated TPA exhibited prolonged *T*_1_ value and sharp ^15^N resonance with a significant chemical shift difference upon complexation with Zn^2+^. The study demonstrated that HP-[^15^N]TPA-D_6_ could detect Zn^2+^ in the low μM range (66 μM) with no interference from protons or other endogenous metal ions. The agent successfully quantified free Zn^2+^ levels in homogenate human prostate tissue and intact human prostate epithelial cells. Given the well-documented significant decrease in total Zn^2+^ levels in malignant prostate tissue, the present findings highlighted the potential diagnostic informativeness of utilizing HP-[^15^N]TPA-D_6_ to measure freely available Zn^2+^ in prostate tissues *in vivo* throughout the progression of prostate cancer.

### DNP sensors for enzymes

**Figure 4.**
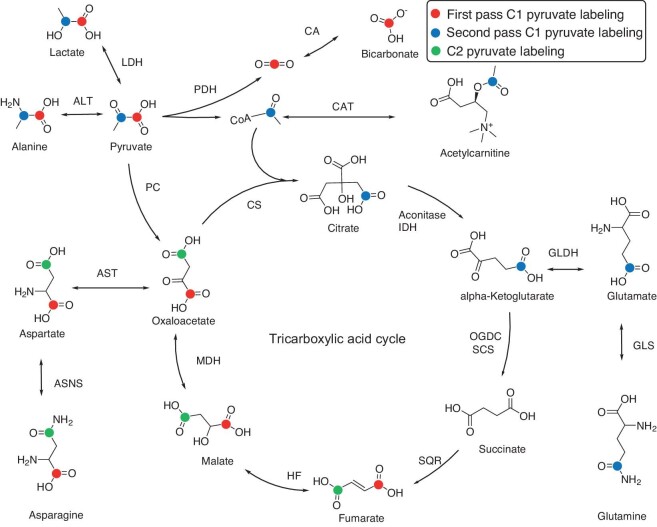
Biochemical labeling resulting from the injection of hyperpolarized (HP) pyruvate is depicted. The labeled carbons, stemming from the metabolism of HP substrates, are highlighted. The red color designates labeling originating from the C1 of pyruvate during the initial metabolic pass. Green denotes the fate of the C1 carbon if it enters through pyruvate carboxylase and progresses backward to fumarate, with a potential label scrambling due to symmetry at this stage. Blue dots indicate labeled intermediates derived from the metabolism of C2 pyruvate. For clarity, TCA (tricarboxylic acid cycle) is abbreviated, with listed enzymes and omitted cofactors. Various abbreviations include LDH (lactate dehydrogenase), ALT (alanine transaminase), CA (carbonic anhydrase), PDH (pyruvate dehydrogenase complex), CAT (carnitine *o*-acetyltransferase), PC (pyruvate carboxylase), CS (citrate synthase), aconitase, IDH (isocitrate dehydrogenase), OGDC (oxoglutarate dehydrogenase complex), SCS (succinyl coenzyme A synthetase), SQR (succinate dehydrogenase), FH (fumarate hydratase), MDH (malate dehydrogenase), AST (aspartate transaminase), GLDH (glutamate dehydrogenase), CoA (coenzyme A), ASNS (asparagine synthetase), and GLS (glutaminase) [91].

The enzymatic transformation of DNP agents is pivotal for the noninvasive visualization of metabolic pathways, encompassing processes such as the uptake/transport kinetics of substrates, their rapid enzymatically-catalyzed conversion, and cofactor availability. [1–^13^C] Pyruvate stands out as the most widely utilized substrate, involving dehydrogenases, redox processes, transaminases, decarboxylases, peptidases, acetyltransferases, acylases, kinases, and hydratases [79]. Prior studies using hyperpolarized NMR have highlighted pyruvate's integral role as a metabolic substrate for *in vivo* monitoring of enzymatic functions [80]. Figure 4 and Table 3 briefly summarize the roles of [1–^13^C] pyruvate in the tricarboxylic acid (TCA) cycle. Comprehensive reviews thoroughly detail the use of hyperpolarized ^13^C agents in preclinical and clinical research [81–83]. In addition to pyruvate, other substrates such as glutamine, asparagine [84–86], and galactose have also been employed as hyperpolarized substrates to study enzyme activities *in vitro* or *in vivo*.

**Table 3.** List of relaxation, polarization, and enzyme response characteristics of reported agents.

**Agents**	***T*_1_/s**	**Nuclei**	**Polarization level/%**	**Sensor**	**Chemical shift difference/ppm**	**Ref.**
C1 of [1,2–^13^C] pyruvic acid	56 (3 T)	^13^C	15.5	PHD	171	[83]
[2–^13^C] pyruvic acid	46 (3 T)	^13^C				[99]
[5–^13^C] glutamine	16 (9.4 T)	^13^C	5	GLS	178.5	[89]
[1–^13^C] ethyl pyruvate	45 (3 T)	^13^C				[85]
C2 of [1,2–^13^C_2_] pyruvic acid	44 (3 T)	^13^C	14.6	PHD	209	[83]
[1–^13^C] glutamine	25 (9.4 T)	^13^C	25	GLS		[88]
[1–^13^C] glutamate	26 (9.4 T)	^13^C	28	GLS		[88]
[1,4–^13^C_2_] fumarate	/	^13^C	26–35	Etoposide	175.4/181.8[1–^13^C]/180.6[4–^13^C]	[86]
^15^N-choline	285 ± 12 (in H_2_O), 120 ± 10 (in human blood) (11.7 T)	^15^N	4.6	Choline Kinase	43.28	[97]
[^15^ N]*L*-Carnitine-*d*_9_	210 (in H_2_O)/160 (*in vivo*) (4.7 T)	^15^N	10	Acetyl-CoA	80	[99]
4-(acetoxy-*d*_3_)-*N,N,N*-tris(methyl-*d*_3_)benzenaminium-2,3,5,6-*d*_4_-^15^N	795 ± 42 in D_2_O, 602 ± 50 in H_2_O (9.4 T)	^15^N		Carboxylesterase	50.5	[100]
[6–^13^C, ^15^N_3_]-arginase	15.13 ± 1.23	^15^N	6.51	Arginase	157	[101]

Chassain *et al.* investigated the uptake of hyperpolarized [1–^13^C] glutamate following a temporary blood-brain barrier (BBB) disruption protocol and its conversion to glutamine in the brain [87,88]. Confirming the BBB disruption protocol, they detected hyperpolarized [1–^13^C] glutamine (175.4 ppm) within the mouse brain and observed the formation of [1–^13^C] glutamine at 174.9 ppm. Their findings suggested that the synthesis of glutamine from hyperpolarized [1–^13^C] glutamine can be monitored *in vivo* in the healthy mouse brain.

Baudin *et al.* developed a method to monitor glutamine-related enzymatic reactions and cellular metabolic processes kinetically [84,89]. They successfully detected the enzymatic reactions of [5–^13^C] glutamine with *L*-asparaginase or glutaminase. Cost-effective MRI systems operating at low-field offer the advantage of portable instrumentation but suffer from a dramatic lack of detection sensitivity. To combat this limitation, Parzy *et al.* introduced a technique for detecting protease-catalyzed hydrolysis of a nitroxide agent via electron-nucleus Overhauser effect using a home-built double resonance system at Earth-field [90]. They observed the kinetics of neutrophil elastase-mediated proteolysis reactions of Suc-(Ala)_2_-Pro-Val-nitroxide enol ester into the ketone form through Earth-field Overhauser-enhanced NMR.

Sando *et al.* evaluated the enzymatic and magnetic properties of g-Glu-[1–^13^C]Gly and developed the deuterated agent, g-Glu-[1–^13^C]Gly-d_2_, which showed a longer *T*_1_ and thus a longer lifespan of the hyperpolarized signal [92]. Their findings confirmed the potential of g-Glu-[1–^13^C]Gly-d_2_ as a novel DNP agent for the detection of GGT, characterized by a longer lifespan of the hyperpolarized signal.

**Figure 5.**
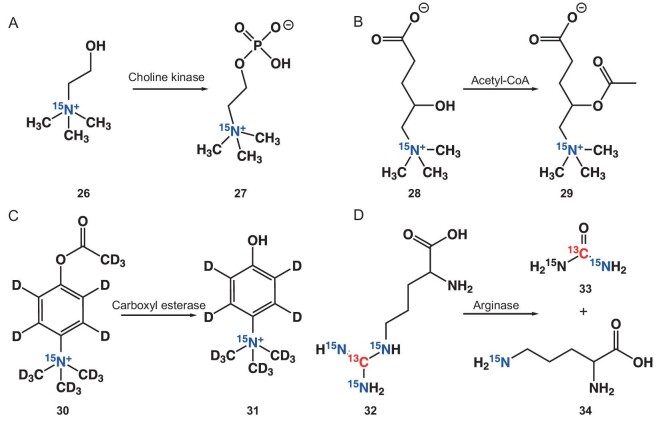
(A) First hyperpolarization experiments of ^15^N-choline. Schematic conversion of ^15^N-choline to ^15^N-phosphocholine [97]. (B) Structures of endogenous *L*-carnitine and its acetylated product. (C) Scheme of carboxyl esterase detection probe reaction. (D) Scheme of arginase detection probe reaction: arginase hydrolyzes arginine to urea and ornithine.

β-Galactosidase is one of the most investigated carbohydrate-converting enzymes. Kjeldsen *et al.* discovered previously unknown intermediates of the lacZ β-galactosidase catalyzed hydrolysis using dDNP NMR [93]. The enzyme lacZ β-galactosidase from *Escherichia coli* was subjected to hyperpolarized substrate, and previously unknown reaction intermediates were observed, including a 1,1-linked disaccharide (Fig. 5). In subsequent research, Kjeldsen *et al.* reported an unexpected anomeric acceptor preference for transglycosylation reactions of β-galactosidases, as revealed through dDNP NMR analysis [94].

The detection of branched-chain α-keto acid dehydrogenase complex (BCKDC) activity is crucial. Park *et al.* demonstrated the feasibility of HP [1–^13^C] α-ketoisocaproate to assess branched-chain-amino-acid aminotransferase (BCAT)/BCKDC activity in F98 glioma *in vivo* [95] Their research revealed an elevation in leucine oxidation within the tumor, distinguishing it as a characteristic metabolic hallmark of glioma. Sando *et al.* have successfully developed a practical DNP-NMR agent to detect aminopeptidase N activity *in vivo* by structure-guided molecular design [96]. The architecturally optimized agent Ala-[1–^13^C]Gly-d_2_-NMe_2_ served effectively for the *in vivo* monitoring of aminopeptidase N activity via DNP-NMR. This report delineated a comprehensive approach for engineering practical, synthetic DNP-NMR molecular agents through structure-guided design.

An exciting advancement in enzyme sensors involves the development of ^15^N-labeled hyperpolarized agents, which facilitate the acquisition of highly valuable information. Numerous examples of ^15^N-labeled chemical sensors have emerged for the detection of enzymatic activity as potential disease biomarkers. Endogenous metabolites, known for their biocompatibility, stand out as ideal candidates for enzyme probes.

Choline (Cho), a naturally occurring molecule in phospholipid metabolism, has gained attention due to its elevated metabolism to phosphocholine (PCho) catalyzed by choline kinases, an established hallmark of cancer. This makes hyperpolarized ^15^N-enriched Cho an ideal tumor imaging substrate, as Gabellieri *et al.* demonstrated, who pioneered *in vitro* choline metabolism to ^15^N-PCho (Fig. 5A) [97]. Cudalbu *et al.* extended this exploration to *in vivo* studies by using MRS on HP ^15^N-Cho to monitor the accumulation of ^15^N-Cho in the mouse brain, revealing the potential of detecting hyperpolarized ^15^N signals *in vivo* [98].

Merritt *et al.* delved into the hyperpolarization and *in vivo* imaging of ^15^N carnitine (Fig. 5B) [99]. Their investigation unveiled the potential of ^15^N-labeled *L*-carnitine as an acetyl-coenzyme A probe for DNP MRI, given its role in acetyl-coenzyme A and fatty acid metabolism. The strategic use of exogenous agents facilitates the flexible development of enzyme probes, enabling the sensing unit to assess specific biological systems. An example of this design strategy is provided by Nonaka *et al.*, who evaluated [^15^N, D_14_]-trimethylphenylammonium (TMPA) to detect carboxyl esterase, an enzyme typically upregulated in various diseases (Fig. 5C) [100]. Remarkably, [^15^N, D_14_] TMPA demonstrated a long spin-lattice relaxation time (1128 s, 14.1 T, 30°C, D_2_O) on its ^15^N nuclei, ensuring prolonged hyperpolarization. This hyperpolarized sensor for carboxylesterase allowed highly sensitive analysis of enzymatic reactions by ^15^N NMR for over 40 min in PBS (pH 7.4, 37°C). In related research, Keshari *et al.* employed hyperpolarized [6–^13^C, ^15^N_3_]-arginine to assess arginase activity *in vivo* (Fig. 5D) [101]. This work reported simultaneous hyperpolarization of cleavable [6–^13^C, ^15^N_3_]-arginine, exemplifying the possibility of dual ^15^N/^13^C labeled HP agents for arginase sensing. Additionally, Sando *et al.* reported Ala-[1–^13^C]Gly-d_2_-NMe_2_ as a DNP-NMR molecular agent for *in vivo* detection of aminopeptidase N activity. Their work outlined a methodical strategy for the development of artificially derived, practical enzyme molecular agents through structure-guided molecular design.

### DNP sensors for redox-oxide

The perturbation of the oxidation/reduction (redox) equilibrium in tissues is intimately associated with the initiation and progression of numerous diseases, emphasizing the potential significance of tissue redox metabolism as a vital parameter for the development of early diagnostic biomarkers [102]. Vitamin C, recognized for its distinct chemical and biological attributes, emerges as an up-and-coming candidate for *in vivo* redox status monitoring. The extended *T*_1_ relaxation time of the C1 carbonyl group, coupled with an oxidation-induced chemical shift difference of ∼5 ppm, and the rapid membrane transport of its oxidized form collectively make vitamin C particularly suitable for this purpose. Comprehensive studies have revealed a variety of cellular processes that mediate the conversion of dehydroascorbate (DHA) back to vitamin C, with pathways delineated that involve glutathione (GSH)-dependent and NADPH-dependent reduction mechanisms [103].

**Figure 6.**
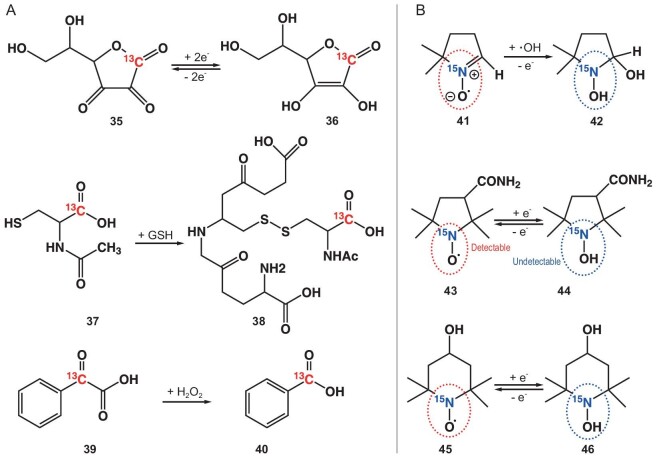
The two redox types of DNP agents. (A) Chemical shift changes of DNP agents before and after the redox reaction. (B) OFF and ON conversion of ESR signal before and after reacting with free radicals.

[1–^13^C] DHA was synthesized to investigate the kinetics of its conversion to vitamin C through GSH-mediated reduction *in vivo*. A study by Wilson *et al.* revealed a swift conversion of [1–^13^C] DHA to [1–^13^C] vitamin C within the kidney, liver, and tumor of a transgenic prostate adenocarcinoma mouse model and in the brain tissue of a normal mouse [104]. Notably, a peak was detected at 19 ± 1.7 s post-injection for the DHA resonance at 174 ppm. Subsequently, a significant metabolite spectral peak at 177.8 ppm was observed, indicative of the *in vivo* formation of [1–^13^C] vitamin C, reaching a maximum of ∼29 ± 1.7 s post-injection. These findings underscore the efficacy of hyperpolarized [1–^13^C] DHA as a sensitive indicator for oxidative and reductive processes in living biological systems. Brindle *et al.* further demonstrated that intravenous administration of DHA elicited a similarly rapid increase in pentose phosphate pathway flux in tumor cells *in vivo*. The hyperpolarization of [1–^13^C] AA and [1–^13^C] DHA was demonstrated at relatively high levels. While the extracellular pool of [1–^13^C] AA underwent rapid oxidation in hypoxic EL4 cell suspensions, its behavior *in vivo* was different [105]. Additionally, the hyperpolarized [1–^13^C] DHA to [1–^13^C] AA conversion has been employed to evaluate resistance to oxidative stress by monitoring the rate of reduction [106]. Chang *et al.* reported a novel hydrogen peroxide (H_2_O_2_) detection method using hyperpolarized ^13^C-MRI, which relies on H_2_O_2_-mediated oxidation of α-keto acids to carboxylic acids [107]. The labeled carboxylate ^13^C1 resonance of the ^13^C-benzoic acid product displayed a chemical shift of 176 ppm, while the unlabeled carboxylate C1 carbon of the initial ^13^C-benzoylformic acid appeared as a doublet at 173.5 ppm. This demonstrated a strong linear correlation with increasing concentrations of H_2_O_2_ (Fig. 6A and Table 4).

**Table 4.** List of relaxation, polarization, and redox-oxide response characteristics of reported agents.

**Agents**	***T*_1_/s**	**Nuclei**	**Polarization level/%**	**Sensor**	**Chemical shift difference/ppm**	**Ref.**
^13^C-DHA (**35**)	56.5 ± 7.6 (3 T)	^13^C	5.9 ± 0.5	e^-^	174.0	[104]
^13^C-Vitamin C (**36**)	29.2 ± 2.5 (3 T)	^13^C	3.6 ± 0.1		177.8/3.8	[104]
^13^C-NAC (**37**)	19.6 (3 T)	^13^C	2.0	GSH	176.8	[108]
^13^C-NAC-GSH (**38**)		^13^C	/		177.4/0.6	[108]
^13^C-BFA (**39**)	24.4 ± 0.4 (11.7 T)	^13^C	5.2	H_2_O_2_	173.5	[107]
^13^C-BA (**40**)		^13^C			175.9/2.4	[107]

Nevertheless, the administration of DHA leads to transient respiratory arrest and cardiac depression in tumor-bearing animals, posing potential safety concerns that may impede its clinical translation. To tackle this issue, Krishna *et al.* present *N*-acetylcysteine (NAC) as a promising and innovative agent for monitoring redox status, effectively addressing the safety concerns associated with dehydroascorbic acid. The researchers successfully developed a stable ^13^C-isotope-labeled NAC and demonstrated its tissue-dependent redox transformation. Notably, NAC formed a disulfide bond in the presence of reduced glutathione, producing a spectroscopically detectable product with a distinct 1.5 ppm shift from the main peak. The biodistribution of hyperpolarized [1–^13^C] NAC and its biochemical transformation during rapid imaging facilitated the monitoring of critical early reactions in thiol biochemistry *in vivo* [108].

The spin-trapping agent 5,5-dimethyl-1-pyrroline *N*-oxide (DMPO) is known for its capability to interact with the hydroxyl radical (·OH) produced in the Fenton reaction, converting it into DMPO-OH, which is identifiable through an EPR spectrum [109]. To illustrate the potential of *in vivo* DNP-MRI for examining liver fibrosis, Murata *et al.* opted for carbamoyl PROXYL as the nitroxide imaging agent to assess the redox status in the liver. Through one-electron transfer reactions, nitroxyl radicals undergo reduction to form the corresponding hydroxylamine or oxidation to generate the corresponding oxoammonium cation species. *In vivo* DNP-MRI imaging of mice was conducted following the intravenous injection of carbamoyl-PROXYL. Signal intensity was enhanced through EPR irradiation without the need for additional heating [110]. In research conducted by Hyodo *et al.*, Tempol was utilized as a nitroxyl radical source to visualize the redox status of the skin. Its remarkably high membrane permeability enabled the exploration of intracellular redox metabolism (Fig. 6B) [111].

## CONCLUSION AND PERSPECTIVES

In recent decades, DNP has proven to be a versatile tool for enhancing sensitivity in liquid NMR. It holds significant relevance in high-resolution NMR spectroscopy within chemistry and biochemistry, with ongoing investigations into its potential applications in biomedical imaging. The tumor microenvironment's influence on tumor growth, invasion, and metastasis is pivotal. This review explores four cancer-associated features within the tumor microenvironment: decreased interstitial pH from increased lactate export, altered metal homeostasis, fluctuating enzyme pathways, and heightened oxidative stress. The dynamic response of DNP agents to biologically relevant concentrations of target molecules and their correlation with specific diseases remain active areas of research. These agents are progressively advancing towards *in vivo* applications for detecting various diseases, offering a swift, noninvasive alternative to ionizing radiation.

Effective design of *in vivo* hyperpolarized DNP agents requires isotope-labeled biomolecules meeting specific criteria: (a) biocompatibility and non-toxicity, (b) availability of an organic synthesis scheme for high-yield production, (c) long spin-lattice *T*_1_ relaxation times, (d) efficient nuclear spin polarization with high substrate concentrations, (e) capability to monitor relevant metabolic pathways or physiological processes, (f) rapid distribution to targeted imaging regions, (g) adequate chemical shift differences between injected substrates and metabolic products, and (h) detectable MR signals in both injected agents and products.

DNP-MRS shows promise in diagnosing tumor microenvironment abnormalities, aiding disease prediction, and guiding personalized treatment. Future clinical trials are poised to utilize reactive DNP agents for imaging lesions, leveraging advancements in hyperpolarization technology to diversify functional MRI applications. This includes the expansion of compliant heteronuclei with long relaxation times, no background signal, and wide chemical shift dispersion. Such developments can potentially revolutionize multiplexing, enabling simultaneous real-time monitoring of multiple physiological processes.

While hyperpolarization significantly improves MRI signals, it is essential to acknowledge that these enhancements are temporary. Swift measurements are crucial to capture the signals before they revert to thermal polarization. Consequently, conducting the entire measurement within a few minutes of creating a hyperpolarized agent faces practical limitations. The implementation of HP-SCA comes with additional challenges, such as low tissue uptake, the requirement for administration at concentrations surpassing physiological levels, and the inability to monitor processes occurring on time scales beyond the hyperpolarized signals’ lifespan. Overcoming these current challenges will demand increased investment in resources and research. Despite these hurdles, we maintain optimistic that as the technique becomes more straightforward and reliable, hyperpolarized dDNP MR could evolve into a standard and potent tool for preclinical research across various disciplines, including oncology, cardiology, hepatology, and neuroscience.
